# Opioid relapse and its predictors among methadone maintenance patients: a multicenter, cross-sectional study in Vietnam

**DOI:** 10.1186/s12954-023-00872-0

**Published:** 2023-09-16

**Authors:** Huong Thi Thanh Nguyen, Dai Xuan Dinh

**Affiliations:** Faculty of Pharmaceutical Management and Economics, Hanoi University of Pharmacy, 13-15 Le Thanh Tong, Hoan Kiem District, Hanoi City, 111000 Vietnam

**Keywords:** Opioid relapse, Concurrent drug use, Associated factor, Social support, Methadone maintenance treatment

## Abstract

**Background:**

Opioid relapse, one of the common and severe problems during methadone maintenance treatment, can give rise to poor treatment outcomes. This study measured the opioid relapse rate and its associated factors among methadone maintenance patients in Vietnam.

**Methods:**

Information about the demographic characteristics and social support of 655 patients was collected through direct interviews. Medical records were used to gather data on treatment characteristics. Relapse was determined via urine opioid test results.

**Results:**

The overall relapse rate of patients during treatment was 13.1%. According to the multivariate logistic regression model, living in mountainous areas (adjusted odds ratio (aOR) = 3.63, 95% CI 1.90–7.46) and long duration of drug use in the past (aOR = 1.06, 95% CI 1.03–1.09) were associated with an increase in the odds of opioid relapse. By contrast, living with many family members (aOR = 0.69, 95% CI 0.55–0.85), having longer treatment time (aOR = 0.80, 95% CI 0.73–0.87), and completely adhering to treatment (aOR = 0.38, 95% CI 0.23–0.64) were protective for opioid relapse. As per the univariate analyses, the odds of opioid relapse declined by 25% for each increase of one close friend or relative (OR = 0.75, 95% CI 0.66–0.86). Regarding social support (range score: 0–100), each additional increase of one score was associated with a 1% decrease in the odds of opioid relapse (OR = 0.99, 95% CI 0.98–0.99). Patient sex, education level, occupation type, patient’s monthly income, family’s monthly income, the number of previous treatments, daily methadone dose, comorbidity, and received antiretroviral therapy were not associated with opioid relapse among patients (*p* > 0.05).

**Conclusions:**

Residence, the role of family and social support, and treatment adherence should be paid more attention to guarantee and enhance the success of methadone maintenance treatment.

## Introduction

Recently, using illicit drugs has been one serious public health problem. According to the United Nations Office on Drugs and Crime, in 2020, roughly 284 million people between the age of 15 and 64 used these drugs at least once. Young people have used more drugs than adults. It was estimated that using these detrimental substances led to 0.5 million deaths and 31 years of healthy life lost. Four drug types commonly used worldwide include cannabis, opioids, amphetamine-type stimulants, and cocaine. In 2020, opioids were used by 1.2% of the global population, doubling the figure for the year 2010. Of an estimated 61 million opioid users, 31 million used opiates, mainly heroin [1]. Notably, opioids were the most lethal group of drugs when about 70% of deaths related directly to drugs were attributed to opioids (mainly overdoses) [1, 2].

Globally, 87 countries had at least one opioid agonist therapy program in 2022 [3]. As per the World Health Organization (WHO), methadone and buprenorphine are two opioid agonists commonly used in clinical practice to manage opioid dependence (for both detoxification and maintenance treatment) [4]. With a solid evidence base, these two therapeutic drugs are featured on the 2021 WHO Model List of Essential Medicines [5]. Compared with buprenorphine, methadone is prioritized in use because of its effectiveness and low cost [4, 6].

In Vietnam, there is an estimation of 235,000 people who use drugs [7]. Vietnam has provided methadone maintenance treatment (MMT) since 2008. During working hours, patients have to go to MMT clinics daily to receive and take methadone under the supervision of medical personnel. During MMT, besides doctors, clinic counselors also bear responsibility for counseling and providing psychosocial support to patients (such as side effects of methadone, how to handle an overdose, healthy lifestyle, and occupation). If the patients do not adhere to the treatment, the MMT can be temporarily stopped and even wholly ceased, and then they can be sent to drug addiction treatment centers [8, 9]. From April 2021, multiday take-home methadone programs have been piloted in some provinces. To be enrolled in these programs, patients must fully comply with MMT. As of September 2022, approximately 51,000 patients took methadone to treat opioid dependence nationwide, including 3,000 patients receiving take-home methadone doses [10].

Opioid relapse is one of the common and severe problems during MMT and can lead to poor treatment outcomes. Concomitant use of opioids was associated with non-adherence and decreased retention rates among MMT patients [11]. The treatment dropout rate increased threefold among patients who continued using drugs during MMT compared with nonusers [12]. Previous studies showed that many patients did not completely abstain from opioids and continued to use these pernicious substances while on MMT [13–19]. After abstinence, resumption of opioid use is one risk factor for opioid-related overdose, which caused 115,000 deaths in 2017 [2]. As a result, concurrent opioid use during MMT should be paid more attention to. To date, negligible data are available on opioid relapse and its associated factors among MMT patients in Vietnam [20–22]. Of these studies, data were collected before the year 2017. Several other limitations included small sample sizes, data collection in only one province, and/or not assessing the role of social support. To fill this knowledge gap, this research investigated opioid relapse while undergoing treatment and determined its predictors among MMT patients in Vietnam.

## Methods

### Ethics

This multicenter, cross-sectional study was assessed and approved by the ethics committee of the Hanoi University of Pharmacy (number: 21-12/PTC-HĐĐĐ). We obtained the permission of clinic directors before interviewing patients and collecting data from medical records. All MMT patients gave their written informed consent before participating in this research.

### Study setting

There is an estimation of more than 51,000 patients enrolling in MMT programs in 330 clinics in 63 provinces/cities all over Vietnam [10]. This study was carried out in one metropolis and two mountainous provinces. Hanoi capital (18 MMT clinics, 4900 patients) is a representative of plain and urban areas, while Son La (13 MMT clinics, 1179 patients) and Dien Bien (9 MMT clinics, 3480 patients) can be considered as mountainous and rural areas. One clinic in Hanoi, one in Dien Bien, and two in Son La were purposely selected to collect data from January to May 2022.

### Participants

Patients were eligible if they (1) were at least 18 years old, (2) were willing to participate in this research and provide written informed consent, (3) were able to communicate and complete the questionnaire, (4) did not contract severe diseases, and (5) have started MMT about more than 3 months at selected clinics. The formula used to compute sample size for estimating a population proportion is *n* = (*Z*/*m*)^2^*.p*.(1 − *p*). With Z = 1.96 (α = 0.05), a margin of error of 3% (m = 0.03), and *p* = 0.134 [21], the minimum sample size was 496. Eligible patients were recruited using a convenience sampling method. Among a total of 1,108 patients from the four selected MMT clinics, 682 patients were approached. 96.04% (655 patients) agreed to participate in this research.

### Data collection

Data collectors were Master’s or specialist students from the Hanoi University of Pharmacy. They were trained about the study’s objectives, the questionnaire, and possible issues during data collection. Eligible patients were approached when they came to MMT clinics to take methadone. Medical staff working in MMT clinics supported the research team in approaching patients. After listening to a short introduction about research aims and procedures, patients were invited to take part in this research and signed written informed consent. Then, they were interviewed by data collectors for about 10–15 min. Information collected via interviews included demographic characteristics of patients and their social support. Patients did not receive any incentives or money for their participation. After the interviews with patients, data collectors continued to gather information on their treatment process from medical records. Data-collection forms will not be shared with anyone to guarantee patient confidentiality. Data were entered into an Excel file with patients’ codes (not including their names).

### Measurement

#### Outcome variables

The primary outcome was whether or not the patients relapsed opioids in the last 3 months. Relapse was determined via urine opioid tests. In Vietnam, as per the treatment guidelines of the Ministry of Health, for the first year of MMT, at least one urine opioid test is performed on a random day per month. After that, urine opioid tests are performed when the doctor requires them (for stable MMT patients, this test can be performed per 3 months) [9]. This explains why a treatment time of at least 3 months was one of the inclusion criteria in this study (to ensure that patients had at least one urine test). In previous studies, relapse events were defined as the use of opioids during MMT, which were determined via patients’ self-report and/or urine test results [13, 23–26]. In this study, a patient was regarded to have relapsed if, in the last 3 months, this person had at least one positive urine test for opioids. In addition, the patient must have had at least one negative test for opioids before this positive test.

#### Demographic characteristics of patients and social support

Demographic characteristics collected through interviews with the patients included: sex, highest education level, place of residence, occupation, patient’s monthly income, family’s monthly income, people living with the patient, and difficulties in MMT. We used the Vietnamese version of the Medical Outcomes Study-Social Support Survey (MOS-SSS) questionnaire to measure patients’ social support. Its validity and reliability were demonstrated [27]. This questionnaire comprises 20 questions. The first question concerns the number of close friends and relatives of the patients. Nineteen remaining questions can be divided into four domains (affectionate support, emotional-information support, positive social interaction, and tangible support) and one additional question. For each question, patients could choose one of the five answers: none of the time, a little of the time, some of the time, most of the time, and all of the time. The social support score of each patient was computed for these 19 questions and transformed to a 0–100 scale. The higher score, the higher the social support [28, 29].

#### MMT characteristics

Patients’ medical records were used to gather information on treatment characteristics. They included patients’ year of birth, initial drug use age, duration of drug use in the past, types of drugs used, routes of drug use, the number of previous treatments, time to start MMT in the current clinic, daily methadone dose, the number of times that patients missed methadone doses in the last month, comorbidity, antiretroviral therapy (ART), and last three urine opioid test results. A patient was classified as “non-adherence” if this person missed at least one methadone dose in the last month.

### Data analysis

R software version 4.2.3 was used to perform statistical analyses. Used packages included *psych, table1, Epi, epiDisplay, FSA, BAS, ResourceSelection, ggplot2, gridExtra,* and *pROC.* Numeric variables (such as social support score) were reported with means (SD—standard deviations) and/or medians (IQR—interquartile range). The *Shapiro–Wilk test* was used to evaluate the normal distribution of numeric variables (a *p* value > 0.05 indicated a normal distribution). Numbers and percentages (%) were employed to describe categorical variables (such as education level). The correlations between two categorical variables were assessed via the *Chi-squared* and *Fisher’s exact tests.*

To determine factors associated with opioid relapse, univariate and multivariate logistic regression models were performed. To minimize the complexity of models and prevent overfitting and multicollinearity, variables in the multivariate logistic regression model were selected using the *Bayesian Model Averaging* method. The *Hosmer–Lemeshow test* and the AUC (area under the curve) were used to assess the goodness of fit of the multivariate model. A *p* value lower than 0.001 was considered statistical significance.

## Results

### Demographic characteristics of MMT patients and opioid relapse

Of the total 655 MMT participants, a majority of them were males (98.5%). About 91.0% had the highest education level of high school or lower. The patients’ average age was 42.43 ± 9.69 years old (median = 43, IQR: 36–49). Nearly two-thirds lived in mountainous areas. A quarter of patients did not work, while nearly half did seasonal or part-time jobs. Farmers and freelancers accounted for 53.7% of participants. The average monthly income of patients and their families was 125.00US$ (median = 84.75, IQR: 0–180.09) and 318.22US$ (median = 211.87, IQR: 169.49–423.73), respectively. In addition, a patient lived with about one to four family members (87.0%) and had one to five close friends and relatives (79.7%). Overall, patients’ average social support score was 63.18 ± 25.73 (median = 65.79, IQR: 47.37–83.55) (Table 1).

**Table 1:** Demographic characteristics of methadone maintenance patients

Demographic characteristics	Number	%	Non-relapse n (%)	Relapse n (%)	*p* value
*Age*					
< 40	254	38.8	212 (83.5%)	42 (16.5%)	0.080
40–50	265	40.5	233 (87.9%)	32 (12.1%)	
> 50	136	20.8	124 (91.2%)	12 (8.8%)	
*Sex*					
Male	645	98.5	561 (87.0)	84 (13.0)	0.628
Female	10	1.5	8 (80.0)	2 (20.0)	
*Highest level of education*					
Primary school or lower	90	13.7	80 (88.9%)	10 (11.1%)	0.618
Secondary school	272	41.5	231 (84.9%)	41 (15.1%)	
High school	234	35.7	205 (87.6%)	29 (12.4%)	
College, university, or higher	59	9.0	53 (89.8%)	6 (10.2%)	
*Area*					
Metropolitan	237	36.2	225 (94.9%)	12 (5.1%)	< 0.001
Mountainous	418	63.8	344 (82.3%)	74 (17.7%)	
*Occupation*					
Not working	164	25.0	146 (89.0%)	18 (11.0%)	0.013
Farmer	150	22.9	123 (82.0%)	27 (18.0%)	
Freelancer	202	30.8	169 (83.7%)	33 (16.3%)	
Trader	46	7.0	42 (91.3%)	4 (8.7%)	
Other occupations	93	14.2	89 (95.7%)	4 (4.3%)	
*Occupation type*					
Not working	164	25.0	146 (89.0%)	18 (11.0%)	0.129
Seasonal or part-time	307	46.9	258 (84.0%)	49 (16.0%)	
Full-time	184	28.1	165 (89.7%)	19 (10.3%)	
*Patient’s monthly income (million Vietnam dongs)*					
< 1	187	28.5	166 (88.8%)	21 (11.2%)	0.303
1–3	248	37.9	209 (84.3%)	39 (15.7%)	
> 3	220	33.6	194 (88.2%)	26 (11.8%)	
*Family’s monthly income (million Vietnam dongs)*					
< 4	143	21.8	121 (84.6%)	22 (15.4%)	0.012
4–7	290	44.3	243 (83.8%)	47 (16.2%)	
> 7	222	33.9	205 (92.3%)	17 (7.7%)	
*Number of family members living with the patient*					
Living alone	38	5.8	33 (86.8%)	5 (13.2%)	0.003
1–2	322	49.2	269 (83.5%)	53 (16.5%)	
3–4	248	37.9	220 (88.7%)	28 (11.3%)	
5 and higher	47	7.2	47 (100%)	0 (0.0%)	
*Number of close friends/relatives*					
None	24	3.7	19 (79.2%)	5 (20.8%)	< 0.001
1–2	262	40.0	210 (80.2%)	52 (19.8%)	
3–4	190	29.0	171 (90.0%)	19 (10.0%)	
5 and higher	179	27.3	169 (94.4%)	10 (5.6%)	
*Social support score*					
0 to < 25	62	9.5	50 (80.6%)	12 (19.4%)	0.040
25 to < 50	109	16.6	88 (80.7%)	21 (19.3%)	
50 to < 75	257	39.2	226 (87.9%)	31 (12.1%)	
75 to 100	227	34.7	205 (90.3%)	22 (9.7%)	

Exchange rate: 1 million Vietnam dongs (VND) = 42.373 US dollars

The opioid relapse rate among patients living in metropolitan areas (5.1%) was significantly lower than that of those living in mountainous areas (17.7%) (*p* < 0.001). Among age groups, the highest rate of opioid relapse was for patients under 40 years old (16.5%). Low relapse rates were found among patients living with at least five family members (0%) and those having more than four close friends/relatives (5.6%). In comparison with the non-relapse group, patients who relapsed to opioid use had fewer family members (mean: 2.50 and 2.02, *p* = 0.005) and fewer close friends/relatives (mean: 4.06 and 2.38, *p* < 0.001), respectively. The average social support score of non-relapse patients (64.52) was also significantly higher than that of the relapse group (54.33) (*p* < 0.001) (Table 1).

### Patients’ treatment characteristics and opioid relapse

Most patients started using drugs at age 30 or lower (85.5%). On average, their duration of drug use was 11.21 ± 7.76 years. Common routes of drug use included injecting (434 patients), snorting/intranasal (172 patients), smoking (145 patients), and oral (6 patients). In the past, fourth-fifths of MMT patients (79.8%) dropped out of treatment at least once. Patients’ average treatment time in the current MMT clinics was 5.04 ± 3.40 years. 85.6% of patients received a daily methadone dose of 120 mg or less. In the last month, the prevalence of complete adherence to treatment among all patients was 73.3%. In addition, there were 350 patients with at least one comorbidity (53.4%). The common comorbidities included hepatitis C (284 patients), Human Immunodeficiency Virus—HIV (70 patients), hepatitis B (64 patients), tuberculosis (17 patients), and peptic ulcer (10 patients). All 70 patients with HIV received ART (Table 2).

**Table 2:** Treatment characteristics and opioid relapse

Treatment characteristics	Number	%	Non-relapse n (%)	Relapse n (%)	*p* value
*Initial drug use age*					
< 20	186	28.4	152 (81.7%)	34 (18.3%)	0.015
20–30	374	57.1	328 (87.7%)	46 (12.3%)	
> 30	95	14.5	89 (93.7%)	6 (6.3%)	
*Duration of drug use in the past (year)*					
< 5	125	19.1	114 (91.2%)	11 (8.8%)	< 0.001
5–10	253	38.6	235 (92.9%)	18 (7.1%)	
> 10	277	42.3	220 (79.4%)	57 (20.6%)	
*The number of previous treatment*					
No	132	20.2	109 (82.6%)	23 (17.4%)	0.259
1–2	329	50.2	290 (88.1%)	39 (11.9%)	
3 or more	194	29.6	170 (87.6%)	24 (12.4%)	
*Comorbidity*					
No	350	53.4	259 (84.9%)	46 (15.1%)	0.206
Yes	305	46.6	310 (88.6%)	40 (11.4%)	
*Antiretroviral therapy*					
No	585	89.3	506 (86.5%)	79 (13.5%)	0.527
Yes	70	10.7	63 (90.0%)	7 (10.0%)	
*Treatment time (year)*					
< 3	196	29.9	150 (76.5%)	46 (23.5%)	< 0.001
3–6	224	34.2	202 (90.2%)	22 (9.8%)	
> 6	235	35.9	217 (92.3%)	18 (7.7%)	
*Daily methadone dose (mg)*					
< 60	238	36.3	203 (85.3%)	35 (14.7%)	0.457
60–120	323	49.3	281 (87.0%)	42 (13.0%)	
> 120	94	14.4	85 (90.4%)	9 (9.6%)	
*Adherence*					
Yes	480	73.3	433 (90.2%)	47 (9.8%)	< 0.001
No	175	26.7	136 (77.7%)	39 (22.3%)	

Patients who commenced using drugs at the age of < 20 years old had a high proportion of opioid relapse (18.3%). Notably, high relapse rates were also found among patients with more than 10 years of drug use (20.6%) and those with less than 3 years of treatment time (23.5%) (*p* < 0.001). Opioid relapse among non-adherent patients (22.3%) was 2.28 times more likely when compared with those who completely adhered to MMT (9.8%) (*p* < 0.001) (Table 2).

### Factors associated with opioid relapse among methadone maintenance patients

**Fig. 1:**
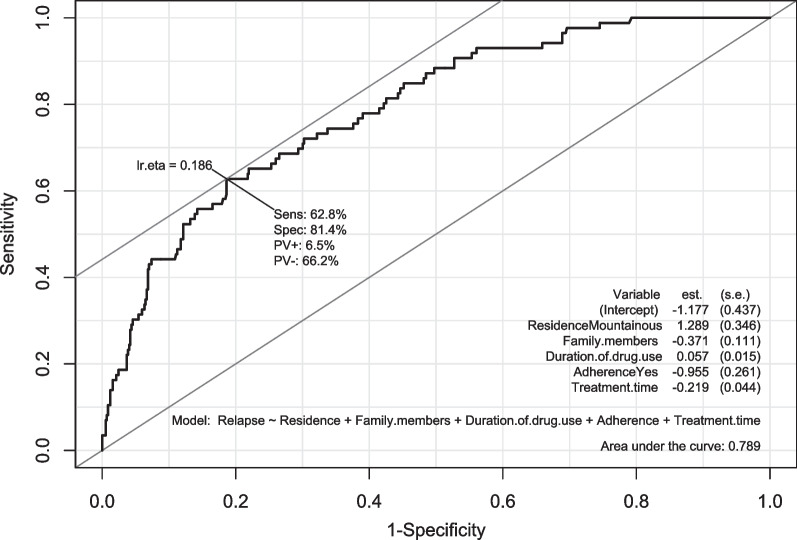
The receiver operating characteristic (ROC) curve analysis for the multivariate logistic regression model. Model: Opioid relapse ~ residence (metropolitan/mountainous) + the number of family members living with MMT patients + duration of drug use in the past (year) + treatment time (year) + treatment adherence (yes/no). The Hosmer–Lemeshow goodness of fit test results demonstrated that the multivariate logistic regression model could adequately fit the data (χ^2^ = 4.742, df = 8, *p* = 0.785). The AUC of this model was 0.789 (95% CI 0.741–0.839)

In the last 3 months, the overall relapse rate during MMT was 13.1%. According to the findings from the multivariate logistic regression model, patients living in mountainous areas were 3.63 times (95% CI 1.90–7.46) more likely to relapse into opioid use as compared to those in metropolitan areas (*p* < 0.001). Patients who completely adhered to MMT experienced a remarkable reduction of 62% in the odds of opioid relapse compared to non-adherent patients (*p* < 0.001). The odds of opioid relapse slightly increased by 6% for each 1-year increase in the duration of drug use in the past (aOR = 1.06, 95% CI 1.03–1.09, *p* < 0.001). In addition, for each one-unit increase in the number of family members living with the patient (one person) and treatment time (1 year), the odds of opioid relapse decreased by approximately 31% (aOR = 0.69, 95% CI 0.55–0.85, *p* < 0.001) and 20% (aOR = 0.80, 95% CI 0.73–0.87, *p* < 0.001), respectively (Table 3, Fig. 1).

**Table 3:** Factors associated with opioid relapse among methadone maintenance patients

Variables	Univariate logistic regression		Multivariate logistic regression	
	OR (95% CI)	*p* value	aOR (95% CI)	*p* value
1. Sex (ref: female)				
Male	0.60 (0.13–2.87)	0.521		
2. Age (years old)	0.98 (0.95–1.00)	0.037		
3. Residence (ref: Metropolitan)				
Mountainous	4.03 (2.14–7.59)	< 0.001	3.63 (1.90–7.46)	< 0.001
4. Education level (ref: College, university, or higher)				
Primary school or lower	1.10 (0.38–3.22)	0.856		
Secondary school	1.57 (0.63–3.88)	0.331		
High school	1.25 (0.49–3.17)	0.638		
5. Occupation type (ref: full-time)				
No	1.07 (0.54–2.12)	0.844		
Seasonal or part-time	1.65 (0.94–2.90)	0.082		
6. Career (ref: Farmer)				
Not working	0.56 (0.30–1.07)	0.079		
Freelancer	0.89 (0.51–1.56)	0.682		
Trader	0.43 (0.14–1.31)	0.139		
Others	0.20 (0.07–0.61)	0.004		
7. Patient’s income per month (million VND)	0.99 (0.92–1.06)	0.749		
8. Family’s income per month (million VND)	0.95 (0.91–1.00)	0.0504		
9. Number of family members living with the patient	0.76 (0.64–0.92)	0.003	0.69 (0.55–0.85)	< 0.001
10. Number of close friends/relatives	0.75 (0.66–0.86)	< 0.001		
11. Social support score	0.99 (0.98–0.99)	< 0.001		
12. Initial drug use age	0.94 (0.90–0.98)	0.004		
13. Duration of drug use in the past (year)	1.06 (1.04–1.09)	< 0.001	1.06 (1.03–1.09)	< 0.001
14. The number of previous treatment	0.94 (0.81–1.08)	0.378		
15. Daily methadone dose (mg)	0.995 (0.99–1.00)	0.064		
16. Treatment time (year)	0.83 (0.76–0.90)	< 0.001	0.80 (0.73–0.87)	< 0.001
17. Adherence (ref: No)				
Yes	0.38 (0.24–0.60)	< 0.001	0.38 (0.23–0.64)	< 0.001
18. Comorbidity	0.73 (0.46–1.14)	0.168		
19. ART (Ref: No)				
Yes	0.71 (0.31–1.61)	0.414		

Exchange rate: 1 million Vietnam dongs (VND) = 42.373 US dollars

*OR* odds ratio, *aOR* adjusted odds ratio, *CI* confidence interval, *ref* reference

As per the results from univariate logistic regression models, the odds of opioid relapse declined by 25% for each increase of one close friend or relative (OR = 0.75, 95% CI 0.66–0.86, *p* < 0.001). Regarding social support (range score: 0–100), each additional increase of one score was associated with a 1% decrease in the odds of opioid relapse (OR = 0.99, 95% CI 0.98–0.99, *p* < 0.001). Furthermore, factors not associated with opioid relapse included the patient’s sex, education level, occupation type, patient’s monthly income, family’s monthly income, the number of previous treatments, daily methadone dose, comorbidity, and ART (*p* > 0.05) (Table 3).

## Discussion

For people using opioids, methadone is one effective maintenance therapy in treating opioid dependence [30–32]. MMT is essential in reducing criminal activities and improving the employment rate and social well-being among patients [33]. During MMT, patients must face many difficulties and challenges [34, 35]. Opioid relapse is a severe problem that can hinder the effectiveness of MMT. In this study, we collected data via interviews with patients and their medical records to investigate opioid relapse and its associated factors among 655 MMT patients in one metropolis and two mountainous provinces of Vietnam. The results showed that the opioid relapse rate was reasonably low. Risk factors for opioid relapse included living in mountainous areas, living with fewer family members, having fewer close friends and relatives, low social support, having a long duration of drug use in the past, having a short treatment time, and not adhering to MMT. These findings can be used to improve patients’ health, thereby contributing to the success of MMT programs in Vietnam.

In this study, most of the patients were males and had a low level of education (high school or lower), in line with the results from previous studies in Vietnam [20–22]. The opioid relapse rate among MMT patients was 13.1%, similar to the finding of a study in the Tuyenquang province of Vietnam (13.4%) [21], but far lower than the results of studies in Iran (76.6%) [36], the United States (54%) [25], and China (44.9%) [13]. The opioid relapse rates varied across areas and countries because of the differences in the study time (year), location (urban or rural, mountainous or metropolitan), the duration used to assess opioid relapse (for example, 3 months, 1 year, or 2 years), and data collection sources (using medical records or direct interviews with patients).

The residence was a factor significantly associated with opioid relapse in this study. The relapse rate of patients living in mountainous areas (Dien Bien and Son La provinces) was significantly higher than those living in metropolitan areas (Hanoi capital). The population density was high in Hanoi (about 2480 people/km^2^) but extremely low in Dien Bien (66 people/km^2^) and Son La (91 people/km^2^) [37]. Low population density and difficulties in traveling can hinder the accessibility to MMT clinics for patients in mountainous areas. In addition, Dien Bien and Son La are two mountainous provinces bordering Laos. In these provinces (especially border areas), geographical barriers and rough terrain can facilitate unlawful activities such as growing poppy plans, drug trafficking, and smuggling [38, 39]. Patients also can be influenced by their peer drug users and relapse into opioid use since many people are using drugs in these two provinces.

The crucial roles of family and social support in treating opioid dependence were displayed in previous studies [40–42]. In China, patients with family members who supported them during MMT were 0.75 times (95% CI 0.60–0.94) less likely to use heroin concurrently. In addition, there was a positive correlation between patients’ family problems and their concurrent heroin use (aOR: 2.01, 95% CI 1.03–3.93) [40]. Patients having no family support (emotion or finance) were 2.03 times more likely to relapse to opioid use in comparison with those having this support (*p* = 0.012) [20]. Social support was a protective factor against opioid relapse for MMT patients in a study conducted in the Sichuan province of China (OR = 0.89, 95% CI 0.83–0.95) [41]. In this study, living with many family members, having many close friends and relatives, and having a high social support score were protective factors against opioid relapse for Vietnamese MMT patients. As a result, during MMT and in patients’ lives, family members, relatives, friends, and the community can support them to reduce stress, overcome discrimination, improve their health-related quality of life, and contribute to lowering the risk of opioid relapse [43].

Treatment adherence was a protector against not only recurrent relapse but also dropout among MMT patients [24, 44]. In China, concurrent opioid use during MMT was associated with poor attendance (the attendance rate was the proportion of days that a patient received methadone over the study period). Patients with an attendance rate of < 20% and from 20 to < 50% were respectively 3.60 (95% CI 1.55–8.33) and 2.80 (95% CI 1.48–5.33) times more likely to experience concomitant opioid use during MMT as compared with those with a rate of ≥ 80% [14]. Like the studies above, non-adherence was a significant risk factor for opioid relapse among Vietnamese patients. Regarding treatment time, a high relapse rate was found among Vietnamese patients with a short duration of MMT, which conformed with the results of previous studies in China [16] and Vietnam [21]. Patients with longer treatment times may understand the pernicious effects of opioids on their health and the benefits of MMT. Therefore, they desired to completely break up with opioids, start a new life, and pursue family happiness [45–47]. Besides, in this study, having a long duration of drug use in the past was an additional risk factor for opioid relapse. Those with a long duration of opioid use may have many peer drug users. Under their influence, MMT patients could be tempted, return to the old drug use environment, and relapse into opioid dependence. Another possible reason is the destructive time-dependent effects of long-term opioid use on the patient’s cognitive abilities, such as impaired neuropsychological function, impulsivity, and deficits in memory, attention, and executive function [48, 49].

Regarding study strengths, instead of interviewing patients or employing a self-reported approach, using medical records to gather information on opioid relapse can help to avoid deliberate concealment and recall bias. A *p* value < 0.001, considered statistical significance, can bring a higher reproducibility of findings. Using the Bayesian Model Averaging method to select independent variables in the multivariate logistic regression model is another strength (for example, reducing the overconfidence and the complexity of models, preventing overfitting and multicollinearity, and giving optimal predictions (beneficial when the target is to make predictions) [50]). However, this research has several following limitations. First, causal relationships between opioid relapse and independent variables cannot be determined in a cross-sectional study. For example, between opioid relapse and treatment non-adherence, we cannot determine which is the cause and which is the effect. In addition, recruiting patients using a convenience sampling technique can lower the generalization of results. Finally, only collecting the data of patients in one city and two provinces cannot be representative of MMT patients all over Vietnam.

## Conclusion

A low rate of opioid relapse was found among Vietnamese MMT patients. Risk factors significantly associated with opioid relapse included living in mountainous areas, living with fewer family members, having fewer close friends and relatives, low social support, long duration of drug use in the past, short treatment time, and treatment non-adherence. In the context of expanding the program of multiday take-home doses of methadone nationwide, the government and the authorities can focus on these factors to support MMT patients during MMT and curb opioid relapse among them.

## Data Availability

The datasets used and/or analysed during the current study are available from the corresponding author on reasonable request.

## Abbreviations

95% CI: 95% Confidence interval
aOR: Adjusted odds ratio
AUC: Area under the curve
HIV: Human Immunodeficiency Virus
IQR: Interquartile range
MOS-SSS: Medical Outcomes Study-Social Support Survey
MMT: Methadone maintenance treatment
ref: Reference
SD: Standard deviation
US$: United States dollar
VND: Vietnam dong
WHO: World Health Organization
